# A 9-month retrospective evaluation of the aetiology and management of patients presenting with encephalitis/meningoencephalitis at a South London hospital

**DOI:** 10.1017/S0950268820000047

**Published:** 2020-02-05

**Authors:** Tehmina Bharucha, Lina Nashef, Nick Moran, Sue Watkins, David Brown, Mark Zuckerman

**Affiliations:** 1 Kings College Hospital, London, UK; 2 Neurosciences, East Kent Hospitals University NHS Foundation Trust, Kent & Canterbury Hospital, Ethelbert Road, Canterbury CT1 3NG, UK; 3 Public Health England, London, UK

**Keywords:** Encephalitis, infectious disease, infectious disease epidemiology

## Abstract

Encephalitis causes high morbidity and mortality. An incidence of 4.3 cases of encephalitis/100 000 population has been reported in the UK. We performed a retrospective evaluation of the diagnosis and management of adults admitted to hospital with a clinical diagnosis of encephalitis/meningoencephalitis. Clinical, laboratory and radiological data were collated from electronic records. Thirty-six patients, median age 55 years and 24 (67%) male were included. The aetiology was confirmed over nine months in 25 (69%) of whom 16 were infections (six viral, seven bacterial, two parasitic and one viral and parasitic co-infection); 7 autoimmune; 1 metabolic and 1 neoplastic. Of 24 patients with fever, 15 (63%) had an infection. The median time to computed topography, magnetic resonance imaging and electroencephalography (EEG) was 1, 8 and 3 days respectively. Neuroimaging was abnormal in 25 (69%) and 17 (89%) had abnormal EEGs. Only 19 (53%) received aciclovir treatment. Six (17%) made good recoveries, 16 (44%) had moderate disability, 8 (22%) severe disability and 6 (17%) died. Outcomes were worse for those with an infectious cause. In summary, a diagnosis was made in 69.4% of patients admitted with encephalitis/meningoencephalitis. Autoimmune causes are important to consider at an early stage due to a successful response to treatment. Only 53% of patients received aciclovir on admission. Neuroimaging and EEG studies were delayed. The results of this work resulted in further developing the clinical algorithm for managing these patients.

## Introduction

In terms of infectious causes, over 100 pathogens have been identified in the aetiology of meningoencephalitis. There are a few national reports defining the epidemiology of encephalitis and there is considerable variation in geographical distribution of infectious causes [1]. An incidence of 4.3 cases of encephalitis/100 000 population has been reported in the UK [2, 3]. This is likely to be an underestimate, with recognised diagnostic and treatment delays contributing to substandard outcomes [4–6]. There are a number of causes of encephalitis, including infectious, post-infectious, autoimmune, inflammatory and neoplastic, however 30–60% of cases remain undiagnosed [2, 7, 8]. The infectious agents responsible are predominantly viruses and bacteria; with herpes simplex encephalitis (HSE) being the most frequently reported infectious aetiology. Amongst the undiagnosed cases, the contribution of autoimmune encephalitis is increasingly acknowledged with improved clinical awareness and access to specialised diagnostic services [9, 10]. Further, infectious aetiologies are still being identified and causative associations being better understood, with access to novel mechanisms for pathogen discovery such as next generation sequencing [11].

Current UK guidelines focus on earlier initiation of aciclovir treatment, within 6 h, carrying out a lumbar puncture (LP) within 12 h and magnetic resonance imaging (MRI) of the brain within 24–48 h [7]. Evidence for the role of aciclovir in improving outcomes in HSE is well-established, however, the limitations in effective treatment for other conditions are striking. Despite evidence based treatment and published guidelines, improving outcomes in encephalitis is an ongoing challenge [12].

We aimed to perform a retrospective evaluation of adult patients with encephalitis in a single tertiary care centre, to analyse the aetiology and outcomes, how these align with current guidelines and identify areas for which clinical management might be improved.

## Methods

A retrospective evaluation of adults with encephalitis or meningoencephalitis admitted to Kings College Hospital from June 27 2013 to February 28 2014. The study included consecutive patients for whom a cerebrospinal fluid (CSF) sample had been collected and received in the virology department during the time period, for which there were available electronic records. All CSF samples underwent testing for HSV types 1 and 2 DNA, VZV DNA, CMV DNA, EBV DNA and enterovirus RNA using in-house multiplex real-time polymerase chain reaction (PCR) assays [3]. A further panel of tests was added if the patients were immunocompromised or had specific imaging that suggested HHV-6 DNA, HHV-7 DNA and JCV DNA testing was required using in-house multiplex real-time PCR assays [3] and HIV-1 RNA using the COBAS AmpliPrep/COBAS TaqMan HIV-1 Test, version 2.0 (TaqMan 2.0). Finally, if there was an indicative travel history, appropriate testing (e.g. for West Nile or Japanese encephalitis) were carried out at, St Louis Encephalitis or tick-borne encephalitis were part of the differential diagnosis, samples were sent to the Public Health England Porton Down reference laboratory. All CSF samples were also tested in bacteriology and CSF gram staining, culture and antimicrobial susceptibility testing, together with latex agglutination testing, and meningococcal and pneumococcal PCR were also performed when indicated.

The case definition for encephalitis was of altered consciousness, (lethargy, irritability, change in personality or behaviour), as well as two of the following: fever, seizures or focal neurological signs, CSF pleocytosis, specific findings on electroencephalography (EEG) or neuroimaging [13]. The Glasgow Outcome Score was assigned from clinical documentation, if recorded, or by the study investigators using electronic records. Data collection and analysis was performed using Microsoft Excel 2013. Ethical approval was discussed with the research ethics facilitator and as this study was observational, retrospective and anonymised, approval was not needed.

## Results

**Fig. 1.**
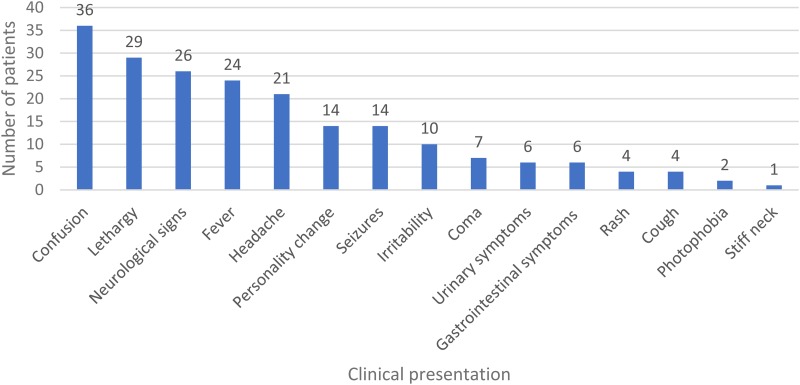
Clinical presentation.

Thirty-six patients were identified with encephalitis/meningoencephalitis over the time period, with a median age of 55 (IQR 44–65) years; and 24 (64%) were male. Ethnicity was 27 (75%) White-British; 7 (19%) Black or Black British-African; 1 (3%) Black or Black British other and 1 (3%) Asian or Asian British Indian. Clinical presentation is demonstrated in Figure 1. The median Glasgow Coma Score was 14 (IQR 10–14). Seven (19%) reported recent infections and seven (19%) reported recent travel abroad. Twelve (33%) had underlying immunosuppression; seven (19%) had HIV and two (6%) had recently received chemotherapy. Data on timing and results of investigations is illustrated in Table 1. Fifteen (42%) patients had an abnormal white cell count (WCC) and 23 (64%) had an abnormal C-reactive protein (CRP).

**Table 1.** Investigations^a^

Peripheral white cell count (median, range)	10 (3–45) 10^9^/l; 15 (42%) abnormal
CRP (median, range)	17 (0–287) mg/l; 23 (64%) abnormal
Imaging abnormal	25 (69%)
Time to CT (median, range)	1 (0–158) days
Abnormal CT	10 (28%)
Time to MRI (median, range)	8 (0–245) days
Abnormal MRI	23 (66%)
Time to EEG (median, range)	3 (0–250) days
EEG abnormal	16 (94%)
Time to LP (median, range)	9 (0–250) days
Autoimmune antibodies tested (included anti-voltage gated potassium channel antibodies; anti-NMDAR antibodies, ANA and ANCA)	35 (97%)

a Calculated from time of review by a medical practitioner in hospital to time of investigation.

An aetiology was identified in 25 (69%) patients. These included 16 (44%) infectious aetiologies of which 7 (28%) were viral, 7 (28%) bacterial and 2 (8%) parasitic. There was a range of pathogens identified including *Neisseria meningitidis*, *Streptococcus pneumonia*, *Listeria monocytogenes*, *Mycobacterium tuberculosis*, *Treponema pallidum*, pyogenic infected shunt, herpes simplex virus-2, human immunodeficiency virus, enterovirus, cerebral malaria and toxoplasmosis. There were also 7 (19%) autoimmune (anti-voltage-gated potassium channel and anti-N-methyl-d-aspartate (anti-NMDAR) antibodies), one (3%) neoplastic, acute disseminated encephalomyelitis (ADEM), central nervous system vasculitis and glioblastoma multiforme. The aetiologies of patients with underlying immunocompromise were largely infectious (8/11, 73%), however it is notable that one of these patients had confirmed anti-NMDA receptor antibody encephalitis. The remaining two with underlying immunocompromise remained undiagnosed. Aetiologies have been stratified in Table 2 as per the system published by Granerod *et al*., in 2010 [3].

**Table 2.** Aetiologies presented in categories

Category	Sub-category	Number	PHE diagnosis
Infection	Bacteria	1	Confirmed *Listeria monocytogenes*
Infection	Bacteria	1	Confirmed *Neisseria meningitidis*
Infection	Bacteria	3	Confirmed *Streptococcus pneumoniae*
Infection	Bacteria	1	Confirmed *Treponema pallidum*
Infection	Bacteria	1	Probable *Mycobacterium tuberculosis*
Infection	Parasite	1	Confirmed cerebral malaria
Infection	Parasite	1	Confirmed *Toxoplasma gondii*
Infection	Virus	1	Confirmed cytomegalovirus
Infection	Virus	1	Confirmed enterovirus
Infection	Virus	1	Confirmed Herpes simplex 2
Infection	Virus	1	Confirmed HIV encephalitis
Infection	Virus	1	Confirmed Varicella zoster virus with HIV
Infection	Virus	1	Probable St Louis encephalitis virus
Infection	Virus and Parasite	1	Confirmed JC virus and *Toxoplasma gondii*
Autoimmune		1	Cerebral vasculitis
Autoimmune		1	Confirmed ADEM
Autoimmune		2	Confirmed vase anti-NMDA receptor antibody encephalitis
Autoimmune		3	Confirmed voltage-gated potassium channel antibody mediated encephalitis
Malignancy		1	Confirmed glioblastoma multiforme
Metabolic		1	Metabolic
Unknown		11	Unknown

Aciclovir was given to 19 (53%) patients on admission. The median duration of aciclovir treatment was 6 (IQR 3–9) days with a range of 0–21. Patients receiving a more prolonged course of 14–21 days fell into the category of either undiagnosed or autoimmune encephalitis. The duration of admission was a median of 32 (IQR 13–59) days. Patient outcome is shown in Table 3.

**Table 3.** Patient outcome at discharge evaluated by the Glasgow Outcome Score

Died	6 (17%)
Persistent vegetative state (Unresponsive for weeks/months or until death)	0 (0%)
Severe disability (Dependent for daily support by reason of mental or physical disability or both)	8 (22%)
Moderate disability (Able to work in a sheltered environment and travel by public transportation)	16 (44%)
Good recovery (Resumption of normal life; there may be minor neurologic and/or psychological deficits)	6 (17%)

## Discussion

The high morbidity and mortality in encephalitis is well-recognised, with challenges in making an impact on outcomes over the last few decades [7, 12].

The demographics derived from these data show important differences compared to other reports from the UK. The median age was much higher at 55 as compared to 30 years, and a relatively high proportion of ethnic minorities, 25%, and immunocompromised patients at 33% as compared to 15% [10]. This is likely to reflect local demographics as well as the setting being a large tertiary referral hospital with specialist neurological, neurosurgical and neuropsychiatric departments.

All patients met the case definition and nearly two thirds presented with headache and fever, and one third with seizures. Fever was seen in patients with both infectious and autoimmune encephalitis. It is important to recognize the latter as effective treatment is available.

In the emergency department, a computed topography (CT) brain scan was carried out within a median of 7 (IQR 1–14) h. However, only 10 (28%) had an abnormal CT and it is recognised that CT is not as sensitive as MRI in identifying brain abnormalities in patients with encephalitis [14]. An MRI, however, was carried out within the 24–48 h time period recommended in current UK guidelines at a median of 14 (IQR 5–23) h. Median time to LP was 12 h, range 1–250, and this is 6 h longer than the recommendation. This is consistent with other reports from the UK of delays in LP, frequently due to the misunderstanding that all patients require brain imaging [15, 16]. However, there are also complex issues in place surrounding workflow of patients in the emergency department, and these are not solved easily. Undoubtedly further work is needed to improve this locally.

A diagnosis was made in 25 (69%) patients, similar to other recent UK studies, and significantly higher than other studies worldwide [10]. This may be due to the expertise and resources available, including the fact that almost all had autoantibody testing. In total 8/11, 73% of the cases remaining undiagnosed had autoantibody testing. However, emerging novel techniques for pathogen discovery such as 16S PCR or next-generation sequencing were not available at that time, and are yet to be validated for the routine testing of patients with encephalitis [17]. Equally, there is ongoing work on point-of-care diagnostic testing that may reduce the time to diagnosis, and implementation of appropriate management [18].

The outcome differed depending on the aetiology: patients with infectious causes had worse outcomes. Patients with infectious aetiologies had a median Glasgow Coma Score of 3 as compared to median 4 of patients with autoimmune disease. There are few data on specific post-encephalitic morbidities in the UK and also on outcomes specific to other causes of encephalitis besides herpes simplex. Survivors of encephalitis may have physical, cognitive, emotional and social difficulties [19]. An outcome study in Sweden reported post-encephalitic epilepsy in 24% of survivors of HSE, representing a 60–90-fold greater risk than the general population. Additionally, a 5–15 and 5–11-fold elevated risk of pulmonary embolism and diabetes mellitus, respectively, were reported. An Austrian study described a high frequency of depressive symptoms among survivors of HSE.

It was concerning that despite guidelines, only 53% received aciclovir. Patients receiving a more prolonged course of 14–21 days fell into the category of either undiagnosed or autoimmune encephalitis. One patient had HSE, but HSV DNA may not be detected in the CSF initially if the LP is carried out too early, or after aciclovir has been given. There is evidence that intrathecal antibody testing may be useful in these cases [17].

In conclusion, it is important to implement standardised first-line investigations to reduce the proportion of cases of unknown aetiology and have standardised diagnostic algorithms. Multidisciplinary work is needed to understand and tackle obstacles in meeting guidelines. In this study, 24% of acute encephalitis was immune-mediated and early recognition of these patients is important as treatment is available and effective, and delayed treatment leads to worse outcomes.
